# Familial Predisposition to Anterior Cruciate Ligament Injury in Australian Rules Footballers

**DOI:** 10.1177/23259671241295613

**Published:** 2024-12-03

**Authors:** Sara Hasani, Julian A. Feller, Kate E. Webster

**Affiliations:** † La Trobe University, Melbourne, Victoria, Australia; + OrthoSport Victoria Research Unit, Melbourne, Victoria, Australia Presented at the AOSSM Annual Meeting, Washington, DC, USA, July 2023; Investigation performed at La Trobe University, Melbourne, Victoria, Australia

**Keywords:** anterior cruciate ligament, Australian football, injury prevention, knee ligaments, medical aspects of sports

## Abstract

**Background:**

A community athlete with an anterior cruciate ligament (ACL) injury is 2.5 times more likely to have a family history of ACL injury than an athlete without an ACL injury. The prevalence of family history and its relationship to ACL injury has not been investigated in elite athletes playing a high-risk sport such as Australian rules football.

**Purpose/Hypothesis:**

The purpose of this study was to determine whether there is an association between primary ACL injury and family history in professional male and female Australian Football League (AFL) players. It was hypothesized that players with a history of ACL injury would have greater rates of family history.

**Study Design:**

Case-control study; Level of evidence, 3.

**Methods:**

All AFL players in the state of Victoria, Australia, were invited to complete a survey querying about their history of ACL injury and whether they had any immediate family members with a history of ACL injury. ACL injury history was compared in those with and without a family history of ACL injury according to sex.

**Results:**

Completed surveys were obtained from 615 out of a possible 672 (91.5%) AFL players, of whom 410 were men and 205 were women. Of players with a history of ACL injury, family history was reported in 47% of male players (15 of 32) and 32% of female players (7 of 22). Male players with an ACL injury history were 3.19 times (95% CI, 1.55-6.76; *P* < .003) more likely to have a positive family history compared with those without ACL injury, and female players with an ACL injury history were 1.7 times (95% CI, 0.66-4.5; *P* = .2) more likely to report a family history than those without.

**Conclusion:**

A strong association was observed between family history and primary ACL injury history in male Australian rules football players. The same association was not statistically significant in female players.

Australian rules football is the most popular form of football in Australia. The premier competition is the Australian Football League (AFL)—an extremely athletic sport with complex agility patterns, multidirectional jumping, and high degrees of contact.^15,16,31^ As a result, players are at high risk for anterior cruciate ligament (ACL) injuries, with 5% of players sustaining an injury during their career.^16^ Most players will sustain their injury with a noncontact mechanism—55% to 60% of cases.^5,24^ Male and female competitions vary significantly in training and competition exposure. Male players are employed full-time across a 23-match season plus finals. The incidence of ACL injuries in the male competition has been around 1 ACL injury per team per season for the past decade.^20,25^ Established in 2017, the inaugural AFL Women (AFLW) competition currently has a 10-match season plus finals, and the athletes compete part-time. When exposure is accounted for, a female AFL player has a 6 times greater risk of ACL injury compared with a male AFL player.^31^

ACL injuries are complex, and the etiology is known to be multifactorial.^26,27^ One risk factor that has received considerable attention is family history.^2,27^ Family history is a complex risk factor with both intrinsic and extrinsic components. Intrinsic factors—such as variations in genetic DNA,^12,13^ narrow intercondylar notch width,^14^ increased tibial slope,^8^ and generalized joint laxity^22,29^—are more prevalent in those with both a family history and a personal history of ACL injury. Extrinsic factors relating to family sporting behaviors—such as higher frequency of physical activity and participation in higher-risk sports—have also been linked to a family history of ACL injury.^1,8^ A 2022 systematic review and meta-analysis^9^ investigated family history and ACL injury in community athletes. Results from the review showed that male and female athletes who had sustained an ACL injury were 2.5 times more likely to also have a family history of ACL injury than those without an ACL injury.^9^ Interestingly, this review also highlighted that the association between family history and ACL injury had not been investigated in an elite sporting cohort.

The primary aim of the present study was to investigate the association between ACL injury in male and female AFL players and a family history of ACL injury. We hypothesized that players with ACL injury history would have higher rates of family history.

## Methods

### Participants

The Australian state of Victoria is home to most AFL clubs (10 of 18). Players from all 10 AFL male and 8 female Victorian clubs were invited to participate in the study. In 2022—the year this study was conducted—there were 2 AFLW seasons. We included players from the first AFLW season (season 6). Medical staff (club physicians or physical therapists) at each club were contacted during the preseason period and briefed on the study's aims, goals, and methodology. Approval for their players to be approached for participation in the study was sought from and provided by the club and their medical staff. All AFL clubs in Victoria agreed to participate in the study. This case-control study was approved by the university human research ethics committee.

### Procedures

**Figure 1.**
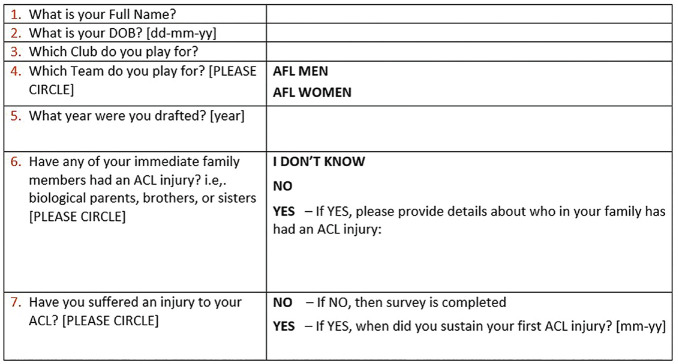
Participant survey. Two further questions regarding ACL reinjuries were part of the survey but were not analyzed for the present study. ACL, anterior cruciate ligament.

Each club was provided with the study materials to give to their players. This included details of the project; individual consent forms; and a participant survey collecting the player's personal, football, and ACL injury information (Figure 1). Survey information and consent were collected in conjunction with club medical staff.

Forms were returned to the research team and the data were entered in a spreadsheet for analysis. If demographic information was missed (eg, date of birth), it was searched for online via the club website. Club medical staff were contacted to provide missing details if a player missed their date of injury or family history details. We ceased data collection at the end of July 2022.

### Outcome Measures

Family history of ACL injury was collected for immediate family members, defined as only first-degree relatives (ie, biological parents, brothers, and sisters).^9^ An ACL history of immediate family members was defined as *yes*, *no*, or *I do not know*. Players who answered *yes* to family history were also asked to list who in their family had sustained an ACL injury. Player ACL injury history was defined as having previously torn an ACL, regardless of whether the injury had occurred when the player was a professional athlete. The dates of injury for all ACL injuries were collected.

### Data Analysis

Statistical analysis was performed using SPSS Statistics Version 29 (IBM). A total of 5 players—4 men and 1 woman—were unsure of ACL injury history in their immediate family members and were thus removed from the dataset. The analysis was completed separately for male and female players, given the differences in the number of training hours, total games, and ACL injury rates. Descriptive statistics were used to summarize the sex, age, and draft year variables. Independent-sample *t* tests were used to compare the age of ACL injury in those with and without a family history. Contingency tables were used to analyze the association between family history and ACL injury using the Crosstabs Risk functions. Statistical significance was defined at *P* < .05. Odds ratios (ORs) with their 95% CIs were calculated.

## Results

### Male Participants

Complete surveys were obtained from 410 of a potential 430 male players (95.3%). At the time of study participation, the mean age (±SD) of the players was 23.6 ± 4.1 years, and they had been listed with an AFL club for a mean of 6.2 ± 4.3 years.

A total of 32 (7.8%) male players reported an ACL injury history, of whom 13 (41%) had sustained their injury before being drafted to an AFL club. The mean age at the time of injury was 20.6 ± 3.8 years. No statistically significant difference was observed in the mean age at the time of injury between those with a family history and those without (mean difference 0.16; *P* > .05).

A total of 97 (24%) male players had a family history of ACL injury. Few players (3%) had ≥1 relative with a family history. Male players with a family history of ACL injury were at 3 times greater odds of having had an ACL injury than their teammates without a family history (OR, 3.19 [95% CI, 1.55-6.76]; *P* < .003) (Table 1).

**Table 1:** The Number of Male Players With Previous ACL Injury With and Without a Family History (n = 410)^*a*^

Variable	Family History, n/Total (%)	OR (95% CI)	*P*
With ACL injury history	15/32 (47)	3.19 (1.55-6.76)	.003
Without ACL injury history	82/378 (22)		

a ACL, anterior cruciate ligament; OR, odds ratio.

### Female Participants

Complete surveys were obtained from 205 of a potential 242 female players (84.7%). At the time of participation in this study, the mean age (±SD) for female participants was 23.8 ± 4.1 years. They had been with a professional AFL club for a mean of 3.4 ± 1.8 years.

A total of 22 (11%) female players reported a history of ACL injury, of whom 9 (41%) reported an ACL injury history and had sustained this injury before being drafted to an AFL club. The mean age at the time of injury was 21.6 ± 4.4 years. No significant difference was found in the age of injury between those with a family history and those without (mean difference, 0.05; *P* > .05).

A total of 46 (22%) female players had a family history of ACL injury. Very few players (2%) reported a family history of ≥1 family member. Female players with a family history were at 1.7 times greater odds (95% CI, 0.66-4.52; *P* = .2) of having sustained an ACL injury than those without (Table 2). The association between a positive family history and ACL injury was not statistically significant in female players.

**Table 2:** The Number of Female Players With Previous ACL Injury With and Without a Family History (n = 205)^*a*^

Variable	Family History, n/Total (%)	OR (95% CI)	*P*
With ACL injury history	7/22 (32)	1.7 (0.66-4.52)	.2
Without ACL injury history	39/183 (21)		

a ACL, anterior cruciate ligament; OR, odds ratio.

## Discussion

The study findings indicated that male Australian rules players with an ACL injury history were 3 times more likely to have a family history than those without (OR, 3.19 [95% CI, 1.55-6.76]; *P* < .003). Within the present sample size, female Australian rules players with an ACL injury history were not significantly more likely to have a positive family history.^28^ To our knowledge, the present study is the first to examine this association in an elite sporting cohort .

Family history is a risk factor that can change from not being present to being present, as a family member may sustain an ACL injury at any given time. The results of this study suggest that regular screening for a family history of injury may be one way to identify athletes who are at a higher risk of ACL injury. This is particularly important, as ACL injuries have the fastest annual growth rate of all knee injuries in young Australians.^17^ Male players previously had a much greater incidence of ACL injuries; however, this gap has narrowed in the past 20 years, which could be attributed to the rise in female participation in “higher risk” sports—such as Australian rules football.^17^ The rate of ACL injuries in the AFL has remained stable over the past decade in the male competition but continues to be of significant concern in the female game.^6,31^ Female-specific anatomic, biological, and hormonal risk factors have been identified as potential contributing factors to the higher rates of ACL injuries in female athletes.^11,23,27^ Some other intrinsic factors—such as neuromuscular control and biomechanics—are modifiable risk factors that have the potential to reduce a female athlete's risk of ACL injury.^21,30^ The discrepancy between male and female ACL injury rates is currently much greater in the AFL than in other high-risk team sports.^6,31^ Many of the current AFLW players did not have a female competitive league to participate in during adolescence. In addition to reduced training and playing history, they did not regularly have access to strength and conditioning or injury prevention programs due to the absence of development programs for female players.^31^ The women's competition is steadily increasing in matches, training time, resources, and quality staff; nevertheless, these sociocultural factors are potentially more significant than family history in female players at this time.^6,7^

ACL injuries have significant consequences for both the player and team, with each player estimated to miss a mean of 12 months from the competition, 23% to 26% of male players not returning to play at the top level, and decreased likeliness of a successful return to the elite level with each subsequent ACL injury.^15,16^ Recurrent ACL injury to the ACL graft or contralateral ACL has been consistently reported in the AFL research at approximately 30% in male players and has not been reported in female players.^15,16,31^ Our study was limited by smaller numbers of ACL injuries, which did not allow us to calculate the risk of subsequent ACL injuries comparing those with and without a family history of ACL injury.^4^ However, the study by Lai et al^15^ audited 15 years of ACL injuries in male AFL players and found that positive family history was associated with an almost 4-fold increased risk of contralateral ACL injury, although they found no association with ACL graft rupture. Their finding reinforces that regular screening of family history would be of benefit and could help identify those at higher risk for subsequent contralateral ACL injury as well as primary ACL injury. In the future, a similar longitudinal audit of female ACL injuries in the AFL would be beneficial.

Injury-reduction programs have been proven to reduce the risk of ACL injury in subelite athletes.^30^ At the elite AFL environment, every athlete completes components of an injury-prevention program—such as strength, plyometrics, and sports-specific agility training—in their weekly training environment.^2^ The injury rate has remained relatively stable despite these interventions. Because of time constraints, these exercises are often delivered to the entire group, potentially at the same time, or given with a performance goal such as improving jump height or reaction speed. It may not be effective to combine programming to suffice both performance and injury prevention at the same time; there is a need to consider a systematic approach to address both demands.^10,18,19^ Ideally, athletes with a family history of ACL injury would receive additional time and specific supervision to complete an individualized program that caters to their strength and biomechanical deficiencies that target high-risk ACL movements.^24^ In Australian rules football, deceleration and side-stepping with defensive pressure have been identified as common mechanisms of ACL injury in both male and female players.^5,24^ Further research is required to investigate the effectiveness of this type of individualized injury-prevention strategy as a complement to an elite program.

This is the first comprehensive study on ACL injury history and prevalence in the AFL, as we successfully surveyed 89% of all the available players in the state of Victoria. Previous studies have reported only outcomes for ACL injuries sustained while on an AFL list.^15,16^ Our study found that 43% of players had sustained their primary ACL injuries before being drafted to an AFL club, and the mean age for ACL injury was subsequently much lower (20.6 years) in this study than previously reported (24 and 23.5 years).^15,16^ This also suggests that other data—such as recurrence rates—may be underrepresented when we consider a player across their lifetime and not just when they are on an AFL list. The totality of the ACL injury problem in this sport has thus not been fully explored.

A population study by Ahn et al^1^ reported that those with a family history of ACL injury were more likely to sustain their injury at a younger age. We found no differences in the age of primary ACL injury for those with and without family history in elite athletes. Recent studies have also noted a greater association with ACL injury in those who have >1 immediate family member with a history of ACL injury.^1,3^ The number of participants with multiple affected family members was too low in this study to appropriately evaluate this association in this elite cohort. Having multiple relatives with ACL injury is a rare occurrence in elite athletes and is unlikely to have practical implications that are different from family history in 1 relative alone.

### Limitations

A limitation of this study was the smaller number of female players compared with male players. Medical staff in the AFLW program reported that it was harder to find the time to survey the players mostly because of the part-time nature of the AFLW program. This potentially contributed to the 78% response rate in female players compared with the 96% response rate in male players. Although it was not a primary aim of this study, the number of ACL injuries was not sufficient to complete any secondary analysis for subsequent ACL injury or investigate the association of injury compared with players with multiple ACL-injured relatives. Another limitation of our study is that we used surveys and did not confirm the accuracy of the reports for those with family history potentially allowing for recall bias. We also did not seek any information around the context of mechanisms for personal or family injuries. Given that 43% of the ACL injuries were sustained before being drafted, we cannot presume that these injuries were sustained while playing Australian rules football. Injuries sustained by contact could be considered unpreventable and may have been useful to exclude from our analysis.^31^ We felt that reporting for injuries without video footage or reporting on behalf of family members could be unreliable; therefore, we chose not to survey for this information.

## Conclusion

This study surveyed a large representative sample of male and female AFL players with regard to their personal ACL history and those of their immediate family members and found a strong association between family history and primary ACL injury history in male players. The same association was not statistically significant in female players.
